# Early Evaluation of Fearfulness in Future Guide Dogs for Blind People

**DOI:** 10.3390/ani11020412

**Published:** 2021-02-05

**Authors:** Fanny Menuge, Míriam Marcet-Rius, Muriel Jochem, Orane François, Camille Assali, Camille Chabaud, Eva Teruel, Justine Guillemot, Patrick Pageat

**Affiliations:** 1 Animal Behavior and Welfare Department, Research Institute in Semiochemistry and Applied Ethology (IRSEA), Quartier Salignan, 84400 Apt, France; m.marcet@group-irsea.com; 2 Frederic Gaillanne Foundation (FGF), 84800 L’Isle-sur-la-Sorgue, France; muriel.jochem@fondationfg.org (M.J.); camille.assali@fondationfg.org (C.A.); justine.guillemot@fondationfg.org (J.G.); 3 Animal Experimentation Service, IRSEA, Quartier Salignan, 84400 Apt, France; o.francois@group-irsea.com; 4 Molecular Biology and Chemical Communication Department, IRSEA, Quartier Salignan, 84400 Apt, France; c.chabaud@group-irsea.com; 5 Data Management and Statistics Service, IRSEA, Quartier Salignan, 84400 Apt, France; e.teruel@group-irsea.com; 6 Head Researcher in Charge for Innovation, IRSEA, Quartier Salignan, 84400 Apt, France; p.pageat@group-irsea.com

**Keywords:** emotional reactivity test, guide dog selection, habituation, puppy raiser, salivary cortisol

## Abstract

**Simple Summary:**

Nearly half of the dogs bred to become guide dogs for blind people fail before the end of their training, the main cause being the presence of fear. Detecting the nature and causes of these fears as early as possible was the main aim of this study. We therefore sought to determine whether the appearance of fear could be explained by insufficient habituation of the puppies. To evaluate fear, we employed an emotional reactivity test (ERT) and a physiological parameter (salivary cortisol). The physiological data supported behavioral data for two of the three parts of the ERT, suggesting that the puppies were able to control their behavioral responses to sound and visual stimuli (SVS). Additionally, the habituation level of the puppies affected both their physiological reactions during the test and their behavioral responses, especially their reactions to SVS. The results suggested that the less accustomed a dog is to a stimulus, the more frightened the dog will be, showing the importance of focusing on early puppy socialization and habituation to improve the numbers of guide dog candidates becoming successful guide dogs.

**Abstract:**

Fear is the leading cause of guide dog failure. Detecting the nature and causes of these fears as early as possible is the first step in preventing their occurrence. The process of habituation is a fundamental part of fear prevention. In this study, 11 puppies, all five months of age, underwent an emotional reactivity test (ERT) composed of 12 scored items, classified into three categories: unknown person (UP), sound and visual stimuli (SVS), and body sensitivity (BS). Salivary cortisol was also measured. Foster families were asked to complete a questionnaire concerning puppies’ habituation. The physiological data were correlated with UP (r = 0.71) and BS scores (r = 0.67), but not with SVS scores (r = 0.16), suggesting the ability of these dogs to control themselves when faced with the latter stimulus category. Additionally, the more time a puppy spent alone, the more likely it was to be afraid of SVS (*p* = 0.05). A correlation, albeit moderate, was detected between cortisol and habituation scores (r = 0.48). These results give us interesting avenues to explore, particularly regarding the importance of focusing on early puppy socialization and habituation to improve the numbers of guide dog candidates becoming successful guide dogs.

## 1. Introduction

According to the World Health Organization’s 2018 figures, nearly 217 million people are visually impaired [1]. In France, 207,000 people live with total or profound visual impairment [2]. Among these people, only 1500 have a guide dog to accompany them during their daily lives [3]. The selection criteria for guide dogs must be strict, requiring a high level of performance from the dog. Indeed, when carrying out their functions, they must be able to control themselves in all circumstances and find solutions when faced with obstacles in order to guarantee the safety of the person they accompany [4,5]. For these reasons, the failure rate of guide dogs is consistently very high worldwide [6,7]. As the cost for one fully trained dog is approximately 25,000 € [8], failures lead to significant financial losses for guide dog organizations. Finding ways to predict the success of a guide dog as early as possible, based on its temperament, in order to help training establishments is therefore an important issue and the subject of many publications [7,9,10,11,12,13,14,15,16,17,18,19,20]. While some dogs are forced out of the program due to physical ailments, behavioral problems account for a majority of guide dog candidates failures [17,21]. Docility, adaptability, stability [7], and passivity [15] are all behavioral traits that facilitate guide dog success. In contrast, fear-related behavior is one of the main reasons for the downgrading of guide dog candidates, behind distraction, shyness, and aggressivity [5,7,17].

Fear is an emotional state caused by an individual’s perception of danger, resulting in an adaptative behavioral response triggered to manage the threat [22]. Fear manifests itself in physiological, behavioral, and emotional ways. An individual’s expression of fear is affected by the interaction of genetic and environmental factors [23], such as inherited characteristics [17], the influence of the present environment, and learned experiences during the sensitive period [24]. During this period, the level of plasticity of the central nervous system allows for the development of individuals based on experiences. Even if opinions on the exact age of the sensitive period and its period of time diverge in the literature, it is commonly accepted that the social development period of puppies is from two to thirteen weeks [25,26,27,28]. For example, focusing on the socialization period, a study of Tiira and Lohi [29] demonstrated that more socialization experiences between eight and twelve weeks of age were associated with lower fearfulness in adult dogs. Emotional reactivity tests (ERTs) are commonly used to score dogs according to their reaction to a series of external stimuli [14]. They can thus be useful for working dog organizations, especially for animal selection, by eliciting not only behavioral but also physiological responses, such as an increase in plasma and salivary cortisol secretion [30,31]. Little consensus exists regarding the tests used, although standardized tests are being developed in the literature [14,20,31]. To meet their own needs, some institutions are even developing their own tests. This is the case for the Frederic Gaillanne Foundation (FGF—L’Isle-sur-la-Sorgue, France), which trains guide dogs specifically for visually impaired children. The FGF administers this test to one-year-old dogs as a selection test just before the beginning of their training. Guide dogs must be able to behave appropriately in any situation, including frightening stimuli [32]. A dog with excessive fear of busy, noisy, or unusual situations would be unsuitable for guide dog training [32].

It has been shown that genetics could influence the development of the dog’s behavior [33]. Besides, these dogs have been selected genetically for their interesting characteristics (e.g., responsive, friendly, clever) that make them potentially good guide dogs [34]. However, habituation and socialization remain primordial in the development of puppies, forming the basis for the prevention of fear [23,24,35,36], and must conform to certain rules to be successful: gradual presentation of the potentially aversive stimulus, regular exposures, and the ability of the animal to freely withdraw from it [37]. To be effective, this process must take place during the sensitive period, in the first months of life [36]. If puppies do not have the opportunity to encounter some types of stimuli during this period, they may develop inappropriate behavioral responses to these stimuli in adulthood [28], which may consist of aggressive or avoidance responses [24]. Notably, a study conducted by Kutsumi et al. [38] showed the benefits of a puppy socialization class for the future temperament of dogs. However, there is no standardized habituation or socialization protocol for future guide dogs. From the age of eight weeks, puppies intended to become guide dogs are usually placed in foster families for approximately one year before beginning their specialized training. It is the foster families’ responsibility to instill the first basics of training but, above all, to socialize the puppies and to accustom them to all types of external stimuli, whether social or environmental [39]. The foster family is therefore a crucial step in the development of future guide dogs [40]. In spite of the importance of this issue, there is a lack of research focusing on the early development of the future guide dogs in their foster families.

This study was carried out in collaboration with the FGF. The ERT developed by its members was used here at an earlier age (at five months of age), and a questionnaire for the foster family measured the amount of habituation that the puppies underwent. The aims were to confirm or refute the validity of this test and to detect the presence of certain fears in the puppies at an early stage. Finally, we also investigated the presence of a link between the level of habituation in the foster family and the puppies’ reactions to the test through behavioral and physiological indicators.

## 2. Materials and Methods

This project was approved by the Research Institute in Semiochemistry and Applied Ethology (IRSEA) Ethics Committee (approval number CE_2020_06_ADOB_03).

### 2.1. Subjects

Eleven 5-month-old dogs from the FGF, including 7 female and 4 male Labernois (Labrador/Bernese mountain dog cross), participated in the study. All subjects were candidate guide dogs and were attributed to a foster family (different for each puppy) at 2 months old. They come from 2 different litters, but they were born to the same breeder.

### 2.2. Procedure

**Figure 1:**
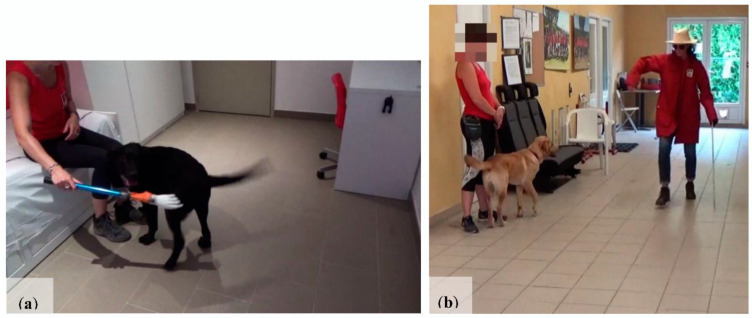
Photos of the rooms used for items 1 to 4 (**a**) and 5 (**b**).

The experiment took place at the FGF. Foster families were asked to bring their puppies between 8:00 and 8:30 a.m. and pick them up in the afternoon. For each puppy, the entire procedure was performed between 9:00 and 11:00 a.m. (2 puppies per morning) to minimize the impact of circadian influences on cortisol levels [41]. This test is an evaluation procedure generally used by the FGF to evaluate dogs at one year of age. It was developed by members of the foundation specializing in canine behavior and by a veterinary behaviorist, adapted from some other procedures used by previously published tests in dogs or other species [20,31,42,43,44]. Due to the young age of the dogs, some items from the original evaluation procedure were removed to avoid overloading them emotionally. Confrontations with joggers, other dogs, and children were therefore eliminated, in agreement with the FFG. These items were placed at the end of the test., therefore, removing them had no effect on the rest of the test. As used in this study, the test, which lasts approximately 20 min, consists of 12 items confronting the animal to different forms of stimulation: sound and visual stimuli (SVS), a test of body sensitivity (BS), and an unknown person (UP), as follows:

1. **Indoor part (4 min)—unfamiliar room (3 m × 3 m) (Figure 1a)**

Item 1 (UP): The dog is alone in the unfamiliar room. A UP enters the room without approaching the dog, stands with his back to the door and his arms at his side, and remains neutral. After 10 s, the UP crouches down and makes contact with the dog. If the dog does not come into contact after 10 s, the UP pats his thighs 3 times to attract the dog. If the dog does not approach, the procedure is stopped and the UP moves on the next item.

Item 2 (BS; UP): A UP sits on the bed in the room; presents an artificial hand on a long arm to the dog for 10 s; and then moves to touch the dog on the back, the sides, the legs, and then the head with the artificial hand. His approach is lateral. If the dog does not approach, the handler calls him once. The handler must not use his actual hands; if the dog retreats, the procedure is stopped.

Item 3 (BS; UP): A UP walks up to the dog, talking to him, and lifts him up from the front. Then, he crouches down, opens the dog’s mouth to look at his teeth, looks inside the ears, takes a front paw and pulls slightly on it, then pulls the tail slightly. If the dog struggles, the procedure is stopped.

Item 4 (UP): The unknown person offers a rope to pull and strongly encourages the dog to play by shaking the toy. He throws the toy approximately 2 m away. If the puppy does not fetch the rope, the UP picks it up and try again, 3 times.

2. **Indoor part (30 s)—corridor (3 m × 12 m) (Figure 1b)**

Item 5 (UP): A handler (a dog trainer who spent one hour with the puppies just before the test—on a walk for 15 min and then on her desk) holds the dog on a leash and stands with his back to the wall in the middle of a corridor. A UP dressed in a strange outfit (hat, glasses, large cape, cane) knocks at the door. He enters and passes in front of the dog, not stopping until the end of the corridor. The UP then turns around, adopts an exaggerated gait (waving his arms, limping), and then crouches down in front of the dog, presenting his hand. After 10 s, the UP gets up and walks away. During this item the handler must not react or move until the stranger is gone.

**Figure 2:**
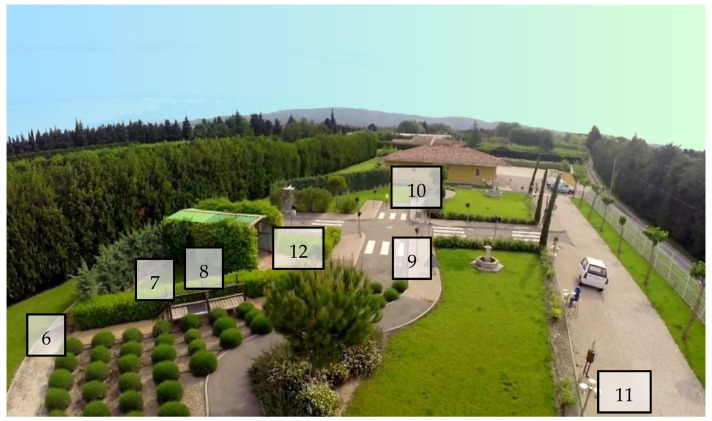
Senses itinerary of the Frederic Gaillanne Foundation (FGF). The locations of the items completed on this course (outdoor part) are indicated by their numbers, from 6 to 12.

3. **Outdoor part (6 min)—senses itinerary: the dog is walked through the course on a 2-m leash by the handler, passing by 8 different points (Figure 2).**

Item 6 (SVS): The handler stops for 1 min in front of a 30 cm high statue representing a dog.

Item 7 (BS): The route continues over a small footbridge. The dog must climb 4 steps (open risers) to cross it. The dog must surmount the obstacle of his own free will. If, despite the handler’s encouragement, the dog refuses to climb the steps, the handler then carries the dog and places it at the top of the footbridge.

Item 8 (BS): The floor of the footbridge has a segment that consists of a metal grid. The dog must walk on it for approximately one meter. As for the stairs, if the dog refuses, the handler carries the dog over this obstacle.

Item 9 (SVS): The handler stops at a given point and waits; a UP moves forward with a wheeled suitcase, passes close to the dog (1 m away), moves 4 m away, comes back towards the dog, and walks away.

Item 10 (SVS): The handler stops at a given point. A UP walks 3 m away from the dog with an umbrella, opens and closes it 3 times at 3-s intervals (left, top, right), and then walks away.

Item 11 (SVS): The handler stops at a given point. At this point, a UP located 2 m away activates a horn 3 times at 3-s intervals.

Items 12 (SVS): The handler stops at a given point. At this point, a UP throws metal cans (3 successively at 3 s intervals) next to the dog (1 m away).

For each item, puppies were scored according to the scale established by the FGF. The addition of all those items gave the ERT overall score. UP score, BS score, and SVS score were also calculated by adding up their corresponding items (see Table 1). Scores vary between −1 and 3. The more the dog shows signs of stress, the lower the score will be. In contrast, the more confident the dog is, the higher the score will be (Table 2). All parts of the ERT were filmed and scored *a posteriori* by two independent observers, specialists in canine behavior.

For relevant test items, additional score was reported as the startle reactions (items 5–6–9–10–11–12) and the avoidance reactions (items 1–2–5–6–7–8–9–10–11–12), scored on a scale of 1–5 points. A high score on the startle reaction scale indicates high startle and high score on the avoidance reaction scale indicates strong avoidance.

We obtained, for each dog, 7 scores in total, recapitulated in Table 1.

In order to analyze cortisol levels, saliva samples were collected just before the test (T0), 15 min afterward (T1), and 45 min afterward (T2) by using the Salivette system (Salivette, Sarstedt, Numbrecht, Germany). For the sake of avoiding contamination, the dogs must not have eaten within 1 h before each sample. As soon as the samples were collected, they were placed in a cooler to keep them at a temperature below 4 °C until they were centrifuged (2 min, 1000 rpm). After being centrifuged, the samples were frozen at −20 °C until analysis (Salimetrics^®^ Cortisol Enzyme Immunoassay Kit, Kiel, Germany).

For the habituation scores, a questionnaire was completed by the foster families to assess the frequency with which the puppies were brought to different locations (unknown places, stores, busy parks, walks in the countryside, along roads highly frequented by cars, public transport, noisy places). For each question, they could choose one of five options (less than once a month—less than once a week—about once a week—more than once a week—every day), giving a global score between 7 and 35. Questions about the frequency (less than once a month—less than once a week—about once a week—more than once a week—every day) and duration (less than 1 h—between 1 and 4 h—between 4 and 7 h—more than 7 h) of time the puppy spent alone at home were also asked, giving a global score between 2 and 9 (isolation score).

### 2.3. Statistical Analysis

The analyses were carried out using SAS 9.4 software Copyright (c) 2002–2012 by SAS Institute Inc., Cary, NC, USA. The significance threshold was fixed at the classical value of 5%.

Interobserver reliability between the 2 independent readers was assessed for each score of the ERT.

Correlations were made between salivary cortisol and ERT scores, between startle and avoidance scores and ERT scores, and between salivary cortisol and foster family questionnaire scores. For this purpose, when normality was verified, the Pearson correlation coefficient was calculated. Otherwise, the Spearman correlation coefficient was used. According to Martin and Bateson [45], r = 0.4–0.7 indicates a moderate correlation (substantial relationship), r = 0.7–0.9 indicates a high correlation (marked relationship), and r = 0.9–1.0 indicates a very high correlation (very dependable relationship).

The associations between the foster family questionnaire score, the ERT score, and the salivary cortisol were also evaluated using linear regressions. The assumptions of normality of model residuals, homoscedasticity, and linearity were verified, and if necessary, the models were simplified to increase power by maximizing the adjusted R^2^.

In order to evaluate the time course of salivary cortisol levels, we applied a general linear mixed model for repeated measures after checking the normality of the residuals.

## 3. Results

The statistical analysis of interobserver reliability revealed strong associations for all the parameters (≥80%). Thus, the average of the data collected by the two readers was taken for the rest of the analysis.

UP, BS, and SVS scores were highly correlated with the ERT overall score (r > 0.8).

A moderate negative correlation was found between salivary cortisol (T1) and the overall score of the ERT (r = −0.54). However, the correlation was higher between salivary cortisol (T1) and BS (r = −0.67) and UP (r = −0.71) scores. A moderate negative correlation was also detected between salivary cortisol (T1) and the habituation score (r = −0.48). Startle behaviors supported the results obtained in the test (see Table 3).

A significant linear regression indicated that the lower the UP score, the more cortisol secreted by the animal (*p* = 0.02). For every five-point increase in the UP score, the average salivary cortisol value at T1 dropped by 0.038 μg/dL. A statistical trend (*p* = 0.0547) was also observed for the effect of the isolation score on the SVS component of the ERT. For every 1-point increase in the isolation score, the average SVS score dropped by 0.854 points.

Concerning the cortisol level, the general linear mixed model for repeated measures revealed no significant difference between before (T0), during (T1), and after (T2) the ERT (GLMM; DF = 2; F = 0.01; *p* = 0.9948) (Table 4).

## 4. Discussion

The aim of this study was to find suitable ways to evaluate puppies at an early stage for the presence of fear, which is the reason for a significant number of failures in guide dog training [32]. The items used to construct this test have been independently validated in the literature [20,31,42,43,44]. The test confronts the animals with three categories of stimuli: sound and visual stimuli (SVS), body sensitivity (BS), and an unknown person (UP). The primary interest was to confirm or refute the validity of this test for use on future guide dogs, i.e., whether it triggered an emotional response in fearful dogs, thus making it possible to detect these fears and highlight undesirable behavior at an early stage. Initially, statistical analyses revealed that all stimulus categories were involved in the test score, as they were all highly correlated with the overall score. When the behavioral data were cross-tabulated with the physiological data, the latter partially agreed with the former. Indeed, salivary cortisol was only moderately correlated with the overall test score, whereas it was significantly correlated with the scores obtained for UP and BS categories. These results suggest that these two categories have a greater impact on cortisol secretion than SVS. The stage at which puppies most readily approach UPs is before two months of age [36]. Results indicate that after this age, mistrust begins to set in. Additionally, evidence suggests that if a puppy has not had contact with strangers during his early development, he may perceive them as something particularly stressful, as previously described [36]. However, another explanation may account for the low correlation between SVS score and salivary cortisol. In one of their studies, Fallani et al. [46] also demonstrated a mismatch between physiological and behavioral data in guide dogs during stressful episodes. In fact, when separated from their owner, this study showed that guide dogs could restrain their behavioral reaction, but their cardiac activation increased. These dogs must learn from an early age to react as little as possible to such stimuli and thus to control their behavioral response. This may explain why direct observation of behavior may not correspond to the animal’s physiological data and may not reflect the dog’s actual experience. The use of physiological parameters is therefore essential to obtain a reliable measurement, especially for guide dogs, as performed in the present study.

The presence and intensity of the startle reaction, which is a brief behavioral response, could, in this case allow more sensitive detection of a fear reaction in the animal. Indeed, the results significantly support the overall score of the test. Startling could therefore be a relevant indicator, as demonstrated in a study conducted by Slabbert and Odendaal [47]. Their research showed that, in police dogs, a significant percentage of puppies that achieved low scores for the startle test did not become successful police dogs. It would therefore be interesting to compare this behavioral response with guide dog training outcomes.

In general, despite the small number of individuals, these results indicate, through the correspondence between behavioral and physiological parameters, that the ERT test influences the emotional state of animals, and thus makes it possible to verify their reactions to different stimuli. These results should be cross-referenced with their entry into the training program (when they are one year old) and their success as a guide dog (at the end of their training) to assess whether this test is an early predictor of whether the dog has the necessary predisposition for this work.

No significant differences were detected among T0, T1, and T2 in salivary cortisol. This could be easily explained by the fact that the puppies were in an unfamiliar environment, separated from their foster families, and in the presence of other dogs, all of which may have increased their cortisol secretion as much as the test itself. Similarly, these same elements explained the fact that the puppies had difficulty returning to a state of homeostasis. This result may suggest the importance of progressive entry into training or prior habituation, as Rooney et al. suggested in one of their studies [48]. This would facilitate dogs’ adaptation to this environment, thus promoting their welfare and their learning capacity [49,50]. Cobb et al. [51] have shown that it is difficult to establish a “normal” interval of cortisol secretion. However, according to the mean salivary cortisol concentration of healthy dogs suggested by some authors [52,53], analysis of salivary cortisol did not detect any abnormally high levels. Abnormally high cortisol would indicate that the dogs were stressed by the program. The results suggest that this was not the case for these dogs.

The questionnaire for foster families gave two scores (habituation and isolation scores). Comparing these with other variables allowed the identification of relationships, which could be the subject of more in-depth studies. For example, a moderate linear relationship was detected between cortisol during the test (T1) and the habituation score. The less the dog is accustomed to frequenting different environments, the more cortisol it will secrete. However, the correlation was low; it would thus be interesting to confirm these results by increasing the sensitivity of the questionnaire and the number of individuals assessed. Similarly, a trend was observed for the effect of the isolation score on the SVS component of the ERT. It is possible that puppies spending more time alone were given less exposure to stimuli. When they encountered unfamiliar stimuli, they showed a fearful response. Although this result was not significant, the strong trend encourages us to believe that a replication of this study in a larger sample of animals would confirm these results at a statistically significant level. According to the answers to the questionnaires, the foster families did not leave the dogs alone for more than four consecutive hours, as recommended by the guide dog schools. The difference therefore lay in the frequency with which this happened, i.e., whether it was a common occurrence. These results showed us the importance of the frequency of these episodes, in addition to their duration. The involvement of foster families is therefore very important in the training process, as the more the puppies accompany them in their daily lives, the more the animals will be able to develop appropriate coping strategies. Accustoming the dogs to any new environment from an early age seems vital in their formation and for the development of an emotional balance essential to their future work, and this difficult role falls on foster families.

## 5. Conclusions

In conclusion, the results showed a correlation between physiological and behavioral data during the test for the UP and BS categories, which shows the good validity of this test for the items belonging to these categories. However, this was not the case for SVS, suggesting a potential ability of the dogs to control their behavior, despite showing physiological signs of stress, when faced with this type of stimulation. Additionally, the results suggested that the less the foster family leaves the puppies alone and the more they accustom the puppies to all kinds of outside stimuli, the less likely they will show physiological signs of stress during ERT.

This study demonstrates the difficulty in providing dogs with an ideal, standardized, and controlled development framework, allowing the acquisition of the necessary basics to promote the emotional balance that is essential for guide dogs. In this context, a plurality of factors could determine the development of fears, and thus the success or failure, of these dogs. These results give us interesting avenues to investigate, particularly on the importance of focusing on the early development period of the puppies, which is a decisive phase influencing their temperament in adulthood. Providing the same environment for the animals would increase the educators’ control over the development of the dogs, especially during the sensitive period, thus minimizing interindividual variation and allowing better targeting and correction of the causes of failure. At this age, ERT could also have a use initially in identifying puppies with a tendency to be fearful, but also to target educational work on desensitizing stimuli, leading to a fear response in these puppies. That could allow more dogs to remain in the program and succeed as a guide dog.

## Figures and Tables

**Table 1:** Scores obtained from the emotional reactivity test (ERT) test associated with the corresponding items.

	UP Score	BS Score	SVS Score	Overall Score	Startle Score	Avoidance Score
Items	1–2–3–4–5	2–3–7–8	6–9–10–11–12	Items 1 to 12	5–6–9–10–11–12	1–2–5–6–7–8–9–10–11–12

**Table 2:** Emotional reactivity test score grid for each item.

Evaluation Criteria	Score
Tail high, ears high, relaxed dog, the dog is at ease, and rather excited or cheerful. The dog remains active, with exploratory behavior.	3
Neutral position: the posture adopted is relaxed. The dog can mark slightly but quickly recovers. The dog has little or no reaction to the stimulus.	2
The tail is low, the dog shows signs of stress (lip licking, yawning, avoidance).	0
The dog is completely panicked (tries to run away) or threatening behavior (grunt, shows teeth).	−1

**Table 3:** Table of correlations between emotional reactivity test (ERT) scores with salivary cortisol (T1), startle and avoidance reaction.

	T1	Startle	Avoidance
Unknown person (UP)	**−0.71** ***p* = 0.02**	**−0.6** ***p* = 0.05**	−0.12 *p* = 0.07
Body sensitivity (BS)	**−0.67** ***p* = 0.03**	**−0.61** ***p* = 0.05**	−0.37 *p* = 0.26
Sound and visual stimuli (SVS)	−0.16 *p* = 0.66	**−0.87** ***p* < 0.01**	−0.45 *p* = 0.16
Overall score	−0.54 *p* = 0.1	**−0.79** ***p* < 0.01**	−0.29 *p* = 0.37

In bold: data with a *p*-value ≤ 0.05.

**Table 4:** Descriptive statistics of salivary cortisol.

Time	N	Mean	Std Error	Minimum	Maximum
T0	11	0.149	0.016	0.077	0.228
T1	10	0.147	0.016	0.065	0.212
T2	9	0.150	0.024	0.091	0.299

## Data Availability

The data presented in this study are available on request from the corresponding author.
